# Pre- and Postoperative Body Composition and Metabolic Characteristics in Patients with Acromegaly: A Prospective Study

**DOI:** 10.1155/2018/4125013

**Published:** 2018-01-08

**Authors:** Xiaopeng Guo, Lu Gao, Xiaodong Shi, Hailong Li, Qiang Wang, Zihao Wang, Wei Chen, Bing Xing

**Affiliations:** ^1^Department of Neurosurgery, Peking Union Medical College Hospital, Chinese Academy of Medical Sciences & Peking Union Medical College, No. 1 Shuaifuyuan, Dongcheng District, Beijing 100730, China; ^2^China Pituitary Disease Registry Center, Peking Union Medical College Hospital, Chinese Academy of Medical Sciences & Peking Union Medical College, No. 1 Shuaifuyuan, Dongcheng District, Beijing 100730, China; ^3^Department of Parenteral and Enteral Nutrition, Peking Union Medical College Hospital, Chinese Academy of Medical Sciences & Peking Union Medical College, No. 1 Shuaifuyuan, Dongcheng District, Beijing 100730, China

## Abstract

**Objective:**

To investigate the preoperative body composition, metabolic characteristics, and postoperative changes in patients with active acromegaly and analyze the effects of gender and age.

**Methods:**

We included 36 patients with untreated acromegaly and 37 patients with nonfunctional pituitary adenomas. Adipose tissue (AT), the visceral fat index (VFI), sclerotin, protein, skeletal muscle, total body water (TBW), intracellular water (ICW), and extracellular water (ECW) were measured using bioelectrical impedance analysis (BIA). Total energy expenditure (TEE) and basal metabolism (BM) were measured with a cardiopulmonary and metabolic analyzer (CMA). Tricep skinfold thickness (TST), bicep circumference, waistline, hipline, and calf circumference were measured with a skinfold caliper and tape. These indices were measured before surgery and 3 months and 1 year after surgery.

**Results:**

Overall, AT, VFI, and TST were lower, whereas sclerotin, protein, skeletal muscle, TBW, ICW, ECW, TEE, and BM were higher in acromegaly patients. Postoperatively, TST rose initially and then decreased, the waistline increased, and sclerotin, skeletal muscle, TEE, and BM decreased. Changes in these indices differed with gender and age in unique patterns.

**Conclusions:**

Body composition and metabolism in acromegaly patients changed after surgery, and gender and age influenced these changes.

## 1. Introduction

Acromegaly is mostly caused by a growth hormone- (GH-) secreting pituitary adenoma, resulting in excess GH and insulin-like growth factor 1 (IGF-1) and thus overgrowth of soft tissue and bone [1, 2]. Although GH has been reported to be the primary driving force, GH and IGF-1 work together to regulate body composition, including glucose, fat, and protein, and to boost basal metabolism. GH is a lipolytic and anabolic hormone that accelerates adipose tissue (AT) breakdown and protein synthesis [3, 4]. In addition to cardiovascular diseases, obstructive sleep apnea-hypopnea syndrome, respiratory dysfunction and neoplasms, body composition, and metabolic changes are also common complications in patients with acromegaly.

Several studies have focused on body composition and metabolic changes in patients with acromegaly. Moller et al. and O'Sullivan et al. demonstrated that the total energy expenditure (TEE) and basal metabolism (BM) were increased in patients with acromegaly compared with the general population [5, 6]. Freda and colleagues were the first to measure the AT distribution with whole-body magnetic resonance imaging (MRI). They found that visceral and subcutaneous AT was significantly reduced in patients with acromegaly compared with the predicted models developed using 315 healthy subjects. The authors also discovered that intermuscular AT increased with acromegaly and concluded that GH-induced insulin resistance might contribute to the increased AT in muscle [3]. Reid et al. [7] studied the relationship between body composition, IGF-1, and insulin resistance using dual-energy X-ray absorptiometry (DXA) and demonstrated that the percentage of the upper limit of normal IGF-1 (%ULN IGF-1) was related inversely to the fat mass and positively to the skeletal muscle. Postoperative changes in body composition in patients with acromegaly have also been explored. Brummer et al. [8] studied body composition using multiscan computed tomography (CT), which indicated that the AT volume increased and the muscle mass decreased after adenectomy. Reyes-Vidal et al. published an article series demonstrating that total, visceral, and subcutaneous AT increased and the skeletal muscle mass decreased after surgery [9, 10]. These changes correlated with the rise in ghrelin, and gender differentially influenced the AT distribution. Fuchtbauer et al. [11] studied the muscle mass and function and verified that patients with acromegaly had increased proximal muscle strength and reduced grip strength. However, both hand grip strength and proximal muscle fatigue increased after biochemical remission.

The most commonly used methods to measure bone mass, AT, lean body mass (LBM), and body water are MRI, DXA, and isotopic dilution [6, 12]. However, MRI is expensive, DXA releases radiation, and these three methods cannot be merged together to evaluate the whole-body mass situation. Thus, more studies are focusing on the application of bioelectrical impedance analysis (BIA). A body composition analyzer (BCA) based on the BIA method can measure and calculate different body compositions, including AT, body water, protein, sclerotin, and muscle, by evaluating resistance and reactance in different body tissues. When the results from the BIA method were compared to standard methods in some studies, the correlation indices were at least 90%. Additionally, the index was 98% when assessing AT and muscle mass using BIA [13, 14]. Patients who underwent BIA avoided receiving unnecessary radiation, and the method was reliable for the determination of the body composition [12–16].

Studies evaluating body composition using the BIA method are rare. Postoperative changes and trends in TEE and BM are unclear. Additionally, whether body composition and metabolism are the same at 3 months and 1 year after surgery is unknown. The effect of age on body composition and metabolism in patients with acromegaly is ambiguous. Thus, this study aimed to prospectively evaluate the preoperative characteristics of body composition and metabolism in patients with untreated acromegaly using BCA and a cardiopulmonary and metabolic analyzer (CMA) to determine the extent of postoperative changes between follow-up at 3 months and 1 year after surgery, and to explore the different effects of age and gender on body composition and metabolism in patients with acromegaly.

## 2. Subjects and Methods

### 2.1. Subjects

This study consecutively enrolled patients with acromegaly admitted to the Department of Neurosurgery at Peking Union Medical College Hospital (PUMCH, Beijing, China) from June 2015 to June 2016 into the experimental group. Each year, approximately 140 patients with acromegaly and over 500 patients with nonfunctional pituitary adenomas were admitted to PUMCH and underwent pituitary tumor resection. The inclusion criteria were as follows: (1) adult patients who presented characteristic clinical manifestations of acromegaly [17, 18]; (2) patients who satisfied the diagnostic endocrine standard [17] (fasting GH > 1 ng/ml, minimum GH > 0.4 ng/ml after oral administration of 75 g of glucose, and a fasting IGF-1 level higher than the age-related reference range); (3) patients with contrast-enhanced MRI of the sellar region indicating a pituitary adenoma; (4) patients who had never received pituitary surgery, irradiation, or medical treatment to reduce the GH and IGF-1 levels; and (5) patients, including patients with gonadotropic hormone substitution, with other pituitary-related hormones at the normal reference levels. Pregnant patients or patients with in-body metal implanted by surgery were excluded. During the same period, patients in our ward with nonfunctional pituitary adenomas matched for gender, age, and body mass index (BMI) were enrolled into the control group. The inclusion criteria were as follows: (1) patients, including patients with gonadotropic hormone substitution, with all pituitary-related hormones at normal reference levels; (2) patients with contrast-enhanced MRI indicating a pituitary adenoma; and (3) patients who had never received pituitary surgery, irradiation, or medical treatment.

Informed consent was obtained from each patient prior to enrollment. This study was approved by the Ethics Committee of PUMCH at the Chinese Academy of Medical Sciences and Peking Union Medical College.

### 2.2. Study Design

All subjects completed a medical history record, physical examination, hormone assays (including fasting GH, fasting IGF-1, oral glucose tolerance test (OGTT) for GH, and other hormone assays), contrast-enhanced MRI of the sellar region, and routine preoperative examinations. Body composition and metabolic measurements were performed for all subjects 1 day before surgery in the Department of Parenteral and Enteral Nutrition of PUMCH. Female participants were required to complete the measurement on days when they were not menstruating. The gender, age, height (to within 0.1 cm), body weight (to within 0.1 kg), BMI, disease duration, and past medical history were recorded before surgery. The disease duration was defined as the interval from the onset of symptoms related to acromegaly to a definitive diagnosis. After excluding the contraindications, all of the patients underwent a transsphenoidal pituitary tumor resection, recovered smoothly, and were discharged 3 days after the operation. Histopathology of the paraffin-embedded tissues and immunohistochemical pathology confirmed the diagnosis of pituitary adenoma.

Patients with acromegaly were followed-up at 3 months and 1 year after surgery in the outpatient service. Acromegaly-related symptoms, pituitary hormones, and pituitary images were recorded. If the patients did not meet the biochemical cure standard, including the presence of residual tumors or neogenetic tumors, and had high GH and IGF-1 levels, we reevaluated their conditions and prepared an optimized treatment. These patients did not undergo body composition and metabolism reassessments. Body composition and metabolism were retested in patients who were cured at the follow-up.

### 2.3. Hormones and Neurological Images

Serum hormones were collected in the Neurosurgery Ward at 6 a.m. after an eight-hour fasting period and sent to the Department of Laboratory Medicine. The OGTT for GH was collected at five time intervals as follows: before, 30 min after, 60 min after, 120 min after, and 180 min after glucose administration. The GH level was measured using an IMMULITE 2000 analyzer with a chemiluminescence assay (L2KGRH2, Siemens Healthcare Diagnostics Products Ltd., UK). The IGF-1 level was measured with a chemiluminescence assay (L2KGFZ, Siemens Healthcare Diagnostics Products Ltd., UK) using the same analyzer. The age-related IGF-1 reference ranges at PUMCH were as follows [19]: 21–25 years, 116–358 ng/ml; 26–30 years, 117–329 ng/ml; 31–35 years, 115–307 ng/ml; 36–40 years, 109–284 ng/ml; 41–45 years, 101–267 ng/ml; 46–50 years, 94–252 ng/ml; 51–55 years, 87–238 ng/ml; 56–60 years, 81–225 ng/ml; and 61–65 years, 75–212 ng/ml. Other pituitary-related hormones, including thyroid stimulating hormone (TSH), triiodothyronine (T3), thyroxine (T4), adrenocorticotrophic hormone (ACTH), cortisol, follicle stimulating hormone (FSH), luteinizing hormone (LH), estradiol, progestin, testosterone, and prolactin, were determined using an ADVIA Centaur XP (Siemens, UK) analyzer with electrochemiluminescence assays (Roche Diagnostics GmbH, Germany). The normal reference ranges were as follows: TSH (0.38–4.34 *μ*IU/ml), T3 (0.66–1.92 ng/ml), T4 (4.3–12.5 *μ*g/dl), ACTH (0–46 pg/ml), cortisol (4–22.3 *μ*g/dl), testosterone (1.75–7.81 ng/ml), and prolactin (0–30 ng/ml). Normal reference ranges of FSH, LH, estradiol, and progestin changed with patients' menstrual cycles.

Hypogonadotropic hypogonadism was characterized by both low gonadal hormones and the presentation of infertility, low libido, or sparse pubic hair. The levels of FSH and LH could be within the normal range or lower. The diagnostic criteria of secondary hypothyroidism included the manifestation of hypothyroidism, a low T3 or T4 level, and low/normal TSH. Adrenal insufficiency was defined as the presentation of weakness and anorexia, low cortisol, and low/normal ACTH.

Contrast-enhanced MRI for the sella was performed using the same machine (3.0 Tesla, Discovery MR750, GE) in the Department of Radiology. The diagnostic radiological presentation for a pituitary adenoma is a solid mass in the sella presented as hypointense on T1-weighted imaging and hyperintense on T2-weighted imaging and an unremarkable reinforcement of the mass.

### 2.4. Somatometry, Body Composition, and Metabolic Measurements

The somatometry indices include tricep skinfold thickness (TST), bicep circumference, waistline, hipline, and calf circumference. The measurement point for the TST is the midpoint between the acromion and the olecranon on the side of the dominant hand. The researcher held the skin and subcutaneous tissue with the thumb, index finger, and middle finger of the left hand and measured the thickness using a skinfold caliper that was accurate to 0.1 mm. The measurement point/plane of the bicep circumference, calf circumference, waistline, and hipline was defined as the midpoint between the acromion and the olecranon on the side of the dominant hand, the plane of the maximal calf circumference, the midpoint between the inferior costal margin and the peak of the iliac crest, and the plane of the maximal hipline, respectively. These measurements were taken three times with a tape and were accurate to 0.1 cm.

Body composition was measured using a BCA (seca mBCA515, Germany) with the BIA method; the indices used included total AT, the visceral fat index (VFI), sclerotin, protein, skeletal muscle, total body water (TBW), intracellular water (ICW), and extracellular water (ECW). Sclerotin indicated the total bone substance of the patients. The basic working principle of the BIA was Ohm's law. A small amount of current was released, and the abovementioned indices were analyzed based on the resistance in different parts of the body [12, 14]. The BCA could calculate TBW and ECW using the impedance values acquired with high-frequency and low-frequency currents. ICW was then calculated using TBW and ECW. Fat mass and fat-free mass were then calculated, and other indices could also be determined by the complex operational formulas inside the machine. Patients were required to walk into the measurement room in the morning after a fasting period with their metal wrist watches, necklaces, finger rings, and earrings removed. After cleaning their palms and the soles of their feet, the patients sat down and relaxed for 5 minutes before the measurement began. The patients stood erect on the measurement table with their bare feet and both hands connected to the electrodes. The internal software of the BCA machine calculated the results in 70 seconds.

The body metabolism was measured with a CMA (COSMED Quark PFT Ergo, Italy); the indices used were the TEE and BM. The patients were required to fast for 12 hours, and smoking was prohibited for 2 hours. The indoor temperature was set at 25 degrees centigrade, and the test cabinet was kept quiet. The patient was placed in a supine position on the test bed with a shield covering the head. The CMA determined the body energy expenditure by calculating the oxygen consumption and carbon dioxide output. Data from the first five minutes were discarded to obtain a more accurate result. The entire process took approximately 20 minutes, and patient movements were prohibited during the test.

### 2.5. Statistical Analysis

We conducted the statistical analysis using the SPSS Statistics software (version 17.0, IBM, USA) and drafted histograms using the Origin software (version 8.0, OriginLab Corporation, USA). Patients in the experimental and control groups were divided into subgroups based on gender and age (>40 years and ≤40 years). Quantitative data were shown as the mean ± standard deviation (SD), and enumeration data were shown as note Levene's test was used to evaluate distributions for the quantitative data; data with normal distributions were assessed using a *t*-test, whereas variables lacking normal distributions were assessed using the Mann–Whitney *U* test. Enumeration data were compared with the *χ*^2^ test. Statistical significance was defined as *p* < 0.05.

## 3. Results

### 3.1. Subjects

Enrolled patients were strictly selected from all admitted patients according to the inclusion criteria. The clinical characteristics of the enrolled patients are presented in Table 1. A total of 36 patients with acromegaly were enrolled into the experimental group (16 males and 20 females). Three patients with acromegaly and seven patients with nonfunctional pituitary adenoma and hypogonadotropic hypogonadism had undergone treatment with estrogen and progesterone substitution. The average age was 45.8 years (SD = 10.2), the BMI was 25.8 kg/m^2^ (SD = 2.7), and the disease duration (DD) was 89.3 months (range from 12 to 276 months). The morbidity due to hypertension was 25%, and to diabetes was 8.3%. A total of 37 patients were enrolled in the control group (15 males and 22 females), with an average age of 43.0 years (SD = 12.9), BMI of 24.8 kg/m^2^ (SD = 4.0), and DD of 27.2 months (range from 1 to 84 months). A total of 8.3% of the patients in the control group were complicated with hypertension and 2.8% with diabetes. The statistical analysis indicated that no significant differences in these indices occurred between the two groups. The GH and IGF-1 levels were significantly increased in the patients with acromegaly and in the subgroup comparisons. Other pituitary hormones exhibited normal reference levels.

Sella MRI showed that the typical radiological characteristics of the tumors were hypointensity on T1-weighted images, hyperintensity on T2-weighted images, and decreased contrast enhancement compared to the surrounding normal pituitary tissue. The average maximum diameter of the tumor in patients with acromegaly was significantly smaller than that in the controls (17.7 ± 7.1 mm versus 24.2 ± 11.6 mm, *p* = 0.005). Invasiveness (Knosp classification > 2) was observed in 25.0% of the patients (9/36) in the experimental group and 29.7% of the patients in the control group (11/37).

Eleven patients with acromegaly were not cured at the follow-up 3 months after surgery. Of these patients, 5 underwent a second surgery and 6 were treated with somatostatin analogues. At the 1-year follow-up, 9 patients were not cured. Of these patients, 4 had a confirmed tumor recurrence and underwent tumor resection, 3 received drug therapy, and 2 underwent three-dimensional conformal radiation therapy. Thus, 25 patients with acromegaly were followed-up with BCA and CMA at 3 months (12 men and 13 women), and 16 patients were followed-up at 1 year (10 men and 6 women) after transsphenoidal surgery.

During the first 3 postoperative months, we identified 2 patients with hypothyroidism, 5 patients with transient adrenal insufficiency (one week after surgery), and 3 patients with hypogonadism. Hormone substitution was applied to help these patients maintain normal pituitary function. At 1 year after surgery, the thyroid function of the 2 patients with hypothyroidism was normal, and euthyrox was withdrawn. Two of the 3 patients with hypogonadotropic hypogonadism recovered to normal gonadal hormone levels without gonadal hormone substitution. Another patient was newly diagnosed with hypogonadism at the 1-year follow-up and subsequently received hormone substitution.

### 3.2. Preoperative Body Composition and Metabolic Characteristics in Patients with Acromegaly

The AT was lower in the patients with acromegaly than in the controls. In acromegaly patients, the VFI was lower (8.73 ± 2.19 versus 10.24 ± 2.45, *p* = 0.007) and the TST was thinner (21.25 ± 7.86 mm versus 24.86 ± 7.90 mm, *p* = 0.045), whereas the bicep circumference, calf circumference, waistline, and hipline revealed no differences. Elements of the body frame, including sclerotin (3.45 ± 0.58 kg versus 3.05 ± 0.42 kg, *p* = 0.001), protein (11.06 ± 2.26 kg versus 9.49 ± 1.64 kg, *p* = 0.001), and the skeletal muscle mass (36.23 ± 7.40 kg versus 30.79 ± 5.33 kg, *p* = 0.001), were significantly larger in the patients with acromegaly. TBW was larger (39.22 ± 8.04 kg versus 33.63 ± 5.82 kg, *p* = 0.001), and its constituents, ICW (25.98 ± 5.39 kg versus 22.45 ± 3.98 kg, *p* = 0.002) and ECW (13.24 ± 2.68 kg versus 11.19 ± 1.86 kg, *p* < 0.001), were both larger in the patients with acromegaly. BM (1535.29 ± 222.26 kg versus 1415.48 ± 205.81 kg, *p* = 0.019) was significantly elevated in the patients with acromegaly.

In the subgroup analysis (Table 2), the differences in body composition and metabolic characteristics seemed more remarkable in the male acromegaly patients and in the acromegaly patients with an age ≤ 40 years. In the female acromegaly patients, although TBW (*p* = 0.039), ECW (*p* = 0.009), sclerotin (*p* = 0.041), protein (*p* = 0.040), and skeletal muscle (*p* = 0.024) were larger than those in the female controls, total AT did not decrease, the AT distribution did not change, and TEE and BM did not increase. Patients with acromegaly aged > 40 years had a lower VFI (*p* = 0.038) and higher skeletal muscle (*p* = 0.031) and ECW (*p* = 0.033) values than those aged > 40 years among the controls, whereas other body composition and metabolic characteristics changed inconspicuously.

### 3.3. Postoperative Changes in Body Composition and Metabolism in Acromegaly

**Figure 1:**
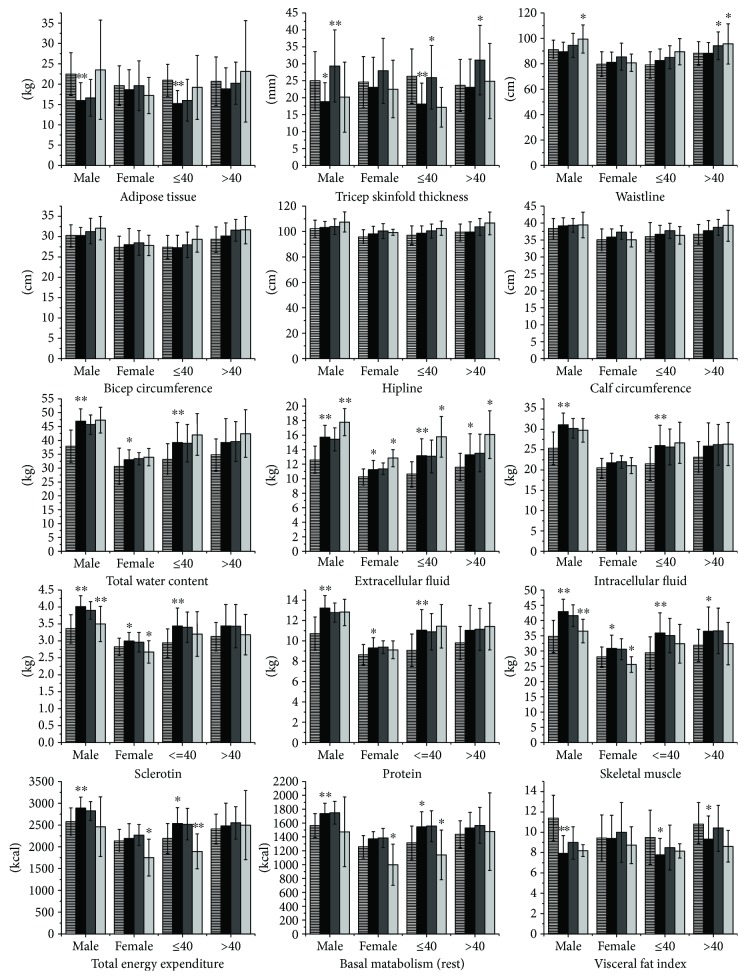
Preoperative characteristics and postoperative changes in body composition and metabolism in patients with acromegaly. This figure includes 15 indices of body composition and metabolism in acromegaly patients and the controls. The bars with transverse lines represent the values of the controls. The black bars represent indices of the patients with untreated acromegaly. Dark gray and light gray bars represent indices of the patients with acromegaly at 3 months after surgery and 1 year after surgery, respectively. The ^∗^ above the black bars represents the preoperative comparison of results between the acromegaly patients and the controls. The ^∗^ above the dark gray and light gray bars represents the comparison of results between indices of the acromegaly patients during the postoperative and preoperative periods. ^∗^*p* < 0.05; ^∗∗^*p* < 0.01.

Among the patients with acromegaly, the body composition and metabolic changes in those who were biochemically cured during the postoperative period are presented in Tables 3 and 4, and the variation trend is illustrated in Figure 1. The total AT, VFI, bicep circumference, calf circumference, and hipline did not change at 3 months and 1 year after surgery. In acromegaly, TST was significantly increased at the 3rd postoperative month in the males and in patients aged > 40 years and even surpassed the controls. However, this measurement deceased again at 1 year after surgery and returned to the preoperative level. Therefore, TST changes during the pre- and postoperative periods presented an interesting and characteristic trend of an “arch bridge.” Although the waistline was similar with that in the controls preoperatively, it was increased during the postoperative period in the male patients and in the patients aged > 40 years. The changes in TST and waistline were not obvious in the females.

As presented in Figure 1, TBW, ICW, and ECW showed trends of slight growth after surgery in the patients with acromegaly, but only the changes in ECW were significant. Although the ECW was obviously higher in the patients with acromegaly before surgery, this measurement was increased 1 year after surgery in all subgroups and was even higher than the preoperative values.

The sclerotin and skeletal muscle mass decreased during the postoperative period in the patients with acromegaly. The decreases were not significant at 3 months after surgery, but they were significantly decreased at 1 year after surgery in both the male and female groups. The protein levels in all four subgroups did not change after surgery.

The TEE and BM decreased to different extents in the patients with acromegaly during the postoperative period. The decrease was significant at the 1-year follow-up, but it was not significant at the 3-month follow-up, especially in the female patients and the patients aged ≤ 40 years.

## 4. Discussion

This study examined the preoperative body composition characteristics and postoperative changes in acromegaly patients using the BIA method and analyzed the surgical effect on changes in metabolism. The preoperative body composition and metabolic characteristics and the postoperative changes in patients with acromegaly presented unique and complex patterns. The total AT, VFI, and TST were decreased preoperatively in the patients with acromegaly and rose again after surgery to different extents. The bicep circumference, calf circumference, waistline, and hipline did not differ from the controls before surgery. However, these measurements all slightly increased postoperatively, and the increase in the waistline in the males and patients aged > 40 years was significant. The TBW, including the ECW and ICW, was higher in the patients with acromegaly before surgery, but the ECW increased 3 months after surgery instead of decreasing. The sclerotin, protein, skeletal muscle, TEE, and BM were higher in the acromegaly patients preoperatively and declined after surgery. Interestingly, gender and age played different roles in influencing the body composition and metabolism. The changes in body composition and metabolism also differed during the different postoperative periods.

Excess GH and IGF-1 can both influence fat metabolism, whereas the effects of GH predominately promote fat breakdown [1, 2]. GH may influence adipogenesis via upregulation of c-Fos, c-Jun, and the STAT family. GH can also upregulate adrenergic receptor b, increase lipase protein expression, and inhibit triglyceride accumulation in the AT by inhibiting the lipase lipoprotein activity [1, 20]. Studies have confirmed that AT is low in patients with acromegaly, although AT can gradually increase after treatment [1, 3, 9, 10, 20].

In this study, gender differences apparently influenced the changes in AT in acromegaly patients. The total AT, VFI, and TST were decreased in the male patients with acromegaly before surgery, whereas the decreases were not significant in the females. Estrogen can inhibit the GH to IGF-1 conversion process in the liver and then slow down lipolysis by suppressing the fat oxidation process [21, 22]. This effect might explain why the AT changes were not as obvious in the female patients with acromegaly. The total AT and TST only decreased in the patients aged ≤ 40 years, which might be attributed to the high GH sensitivity of peripheral AT. The GH sensitivity of AT decreases with age, and TEE and BM simultaneously decline; therefore, AT was not significantly reduced in the patients aged > 40 years compared to the controls [22]. The postoperative “arch bridge” trend of TST is unique and interesting, indicating that the GH sensitivity of subcutaneous AT is relatively higher at 3 months after surgery, resulting in reexpansion. However, TST decreased again at 1 year after surgery, which deviated from the original increasing trend. Whether a recall mechanism exists in the subcutaneous AT to force TST to return to its long-term preoperative status needs to be determined.

The bicep circumference, calf circumference, waistline, and hipline of the patients with acromegaly accorded with those of the controls regardless of gender and age. This phenomenon indicated that apart from enlarged fingers, toes, and facial changes, the body shape did not obviously change in the patients with acromegaly. This finding might be the comprehensive outcome of decreased body fat and increased sclerotin and skeletal muscle. Although the bicep circumference, calf circumference, waistline, and hipline in the male patients had a tendency for a moderate increase, only the increase in the waistline in the males was significant 1 year after surgery.

One essential element of the material basis of body anabolism is water. Excess GH in patients with acromegaly accelerates the retention of water and sodium to ensure the normal proceeding of boosted anabolism [23, 24]. The retention of water and sodium in acromegaly is realized by GH activating the renin-angiotensin-aldosterone system and thereby promoting atrial natriuretic factor, prostaglandins, and nitric oxide production [24]. Clinical studies have demonstrated that the primary type of body water retention is ECW retention [1, 6]. Our results were in line with this viewpoint. Additionally, this study for the first time systematically focused on changes in TBW, ECW, and ICW using the BIA method during different postoperative periods. Although TBW, ECW, and ICW all increased after surgery, the TBW and ICW changes were not significant, whereas the ECW increased obviously with the decline in GH. In O'Sullivan et al.'s study, the administration of somatostatin (octreotide) for 3 months resulted in a decrease in the ECW of 2 kg [6]. Hansen et al. [25] demonstrated that administration of octreotide for 4 weeks led to a significant reduction in the ECW. The body water seemed to decrease with the decline in GH. However, our results showed an upward trend for ECW after surgery in contrast to the downward trend observed shortly after surgery in the literature. Two possible reasons may account for this discrepancy. One is the short follow-up period of the previous studies, and the other is that the BIA method was not previously confirmed to be reliable for evaluating body water in acromegaly patients in the literature. Prospective studies concerning the reliability of BIA for body water, the differences between BIA and DXA, and their possible mechanisms need to be explored.

The effect of excess GH on the acceleration of anabolism results in an increase in the LBM in untreated acromegaly, and the LBM decreases with the decline in GH [24, 26–29]. GH can promote protein synthesis and increase the skeletal muscle mass by inhibiting myostatin in the localized muscle [24, 26]. In this study, sclerotin, protein, and skeletal muscle were higher in the acromegaly patients before surgery. However, only the sclerotin and skeletal muscle decreased during the postoperative period. The decrease in the skeletal muscle mass may explain why most patients in clinics experience fatigue during the postoperative period. In 2017, Fuchtbauer and his colleagues also addressed this question by demonstrating that proximal muscle fatigue and grip strength increased after biochemical remission in patients with acromegaly [11]. Regarding the bone changes, Vestergaard and Mosekilde found that GH promoted bone mineral accumulation and that the osteoporosis and fracture rates were low in patients with untreated acromegaly [30]. Kaji et al. [29] found that the bone mineral density was increased in patients with acromegaly compared to the general population. Our study indicated that sclerotin decreased after surgery in patients with acromegaly, which established a basis for postoperative research on bone mineral density and the osteoporosis and fracture rates in acromegaly patients from a body composition perspective.

The BM has been reported to be slightly increased in patients with acromegaly and in patients receiving GH replacement therapy [27]. An elevated metabolic rate was positively correlated with increased LBM, which might be caused by increased activity of the Na+/K + −ATPase. Excess GH promoted anabolism when the oxygen consumption was elevated and resulted in enhanced glucose metabolism. All of these factors contributed to the elevated BM [27, 31]. A study by Salomon et al. found that the average BM for patients with acromegaly was 8201 kJ, which was significantly higher than the controls [27]. Few studies have focused on the effects of gender and age on energy expenditure in patients with acromegaly. In this study, the preoperative increases in TEE and BM were obvious in the males, whereas the decrease at 1 year after surgery was significant in the female patients. For patients with acromegaly aged ≤ 40 years, the preoperative increase and postoperative decrease in TEE and BM were both typical.

In conclusion, the body composition (body fat, water, sclerotin, muscle, and protein), body metabolism (TEE and BM), and postoperative changes in patients with acromegaly presented interesting and unique patterns. The BIA method can systematically and comprehensively evaluate these changes. The characteristics can help to perform better preoperative assessments and to assuage patients' doubts and improve their quality of life. The mechanism underlying gender-specific and age-related body composition and metabolic characteristic changes during the pre- and postoperative periods is still ambiguous and needs to be verified.

## Figures and Tables

**Table 1:** Clinical characteristics of the acromegaly patients and the controls.

		Male	Female	Age ≤ 40	Age > 40
Height, cm	Acromegaly patients	172.99 ± 4.81^∗∗^	160.04 ± 6.45	166.84 ± 7.39	165.13 ± 9.50
	Controls	165.37 ± 8.49	161.12 ± 5.70	162.04 ± 5.52	163.46 ± 8.30

Weight, kg	Acromegaly patients	79.81 ± 9.24	64.30 ± 7.81^∗^	69.51 ± 10.54	72.26 ± 12.09
	Controls	74.35 ± 9.76	60.22 ± 9.90	62.58 ± 13.02	68.51 ± 10.75

BMI	Acromegaly patients	26.61 ± 2.23	25.13 ± 2.95^∗∗^	24.90 ± 2.63	26.35 ± 2.69
	Controls	27.28 ± 3.71	23.15 ± 3.24	23.75 ± 4.27	25.64 ± 3.60

Hypertension, *n*/%	Acromegaly patients	3/18.75%	6/30.00%	0/0.00%	9/40.91%
	Controls	1/6.67%	2/9.09%	0/0.00%	3/14.29%

Diabetes mellitus, *n*/%	Acromegaly patients	2/12.50%	3/15.00%	2/14.29%	3/13.64%
	Controls	1/6.67%	0/0.00%	0/0.00%	1/4.76%

Fasting GH, ng/ml	Acromegaly patients	32.71 ± 45.08^∗^	14.54 ± 15.31^∗∗^	30.93 ± 48.12^∗^	17.32 ± 17.16^∗∗^
	Controls	0.47 ± 0.49	0.47 ± 0.45	0.62 ± 0.48	0.36 ± 0.42

IGF-1, ng/ml	Acromegaly patients	1040.03 ± 179.60^∗∗^	785.35 ± 239.15^∗∗^	1007.29 ± 289.03^∗∗^	829.34 ± 248.12^∗∗^
	Controls	179.60 ± 127.48	164.23 ± 92.72	194.00 ± 135.01	152.52 ± 77.82

Comparisons were performed between the patients with acromegaly and the controls. ^∗^*p* < 0.05; ^∗∗^*p* < 0.01.

**Table 2:** Differences in the body composition and metabolism before surgery between the acromegaly patients and the controls.

	Male		Age ≤ 40		Female		Age > 40	
	Acromegaly patients	Controls	Acromegaly patients	Controls	Acromegaly patients	Controls	Acromegaly patients	Controls
Bicep circumference, cm	30.26 ± 1.93	30.18 ± 2.65	27.29 ± 3.01	27.39 ± 2.82	27.96 ± 3.99	27.24 ± 2.84	30.06 ± 3.25	29.23 ± 3.12
Tricep skinfold thickness, mm	18.94 ± 5.69^∗^	25.03 ± 8.71	18.25 ± 6.11^∗∗^	26.41 ± 8.11	23.10 ± 8.95	24.75 ± 7.51	23.16 ± 8.37	23.69 ± 7.72
Calf circumference, cm	39.11 ± 2.35	38.20 ± 3.07	36.57 ± 2.65	35.85 ± 4.24	35.80 ± 2.42	34.97 ± 3.23	37.72 ± 2.99	36.60 ± 2.91
Waistline, cm	89.48 ± 7.40	90.97 ± 7.46	82.36 ± 9.22	78.96 ± 10.23	80.87 ± 8.27	79.46 ± 9.91	86.18 ± 8.59	88.06 ± 9.23
Hipline, cm	102.94 ± 4.84	102.15 ± 6.79	98.52 ± 5.20	96.93 ± 7.20	97.96 ± 5.96	95.44 ± 5.72	101.22 ± 6.30	99.09 ± 6.77
Adipose tissue, kg	15.93 ± 4.40^∗∗^	22.47 ± 5.24	15.22 ± 3.26^∗∗^	20.97 ± 3.93	18.64 ± 4.89	19.64 ± 4.86	18.84 ± 5.16	20.64 ± 6.00
Visceral fat index	7.92 ± 1.78^∗∗^	11.41 ± 2.26	7.78 ± 1.66^∗^	9.51 ± 2.69	9.39 ± 2.31	9.44 ± 2.28	9.34 ± 2.30^∗^	10.80 ± 2.15
Sclerotin, kg	4.02 ± 0.32^∗∗^	3.36 ± 0.42	3.45 ± 0.53^∗∗^	2.94 ± 0.42	3.00 ± 0.26^∗^	2.83 ± 0.26	3.45 ± 0.63	3.13 ± 0.42
Protein, kg	13.23 ± 1.25^∗∗^	10.71 ± 1.65	11.06 ± 2.04^∗∗^	9.08 ± 1.61	9.32 ± 1.01^∗^	8.65 ± 1.00	11.05 ± 2.44	9.80 ± 1.63
Skeletal muscle, kg	42.94 ± 4.09^∗∗^	34.78 ± 5.32	35.89 ± 6.68^∗∗^	29.41 ± 5.27	30.86 ± 4.40^∗^	28.07 ± 3.28	36.45 ± 7.97^∗^	31.85 ± 5.25
Total body water, kg	46.93 ± 4.47^∗∗^	37.97 ± 5.82	39.22 ± 7.28^∗∗^	32.14 ± 5.73	33.05 ± 3.63^∗^	30.67 ± 3.57	39.21 ± 8.66	34.76 ± 5.75
Intracellular water, kg	31.19 ± 2.89^∗∗^	25.38 ± 3.99	26.04 ± 4.98^∗∗^	21.52 ± 4.02	21.80 ± 2.37	20.45 ± 2.48	25.93 ± 5.75	23.15 ± 3.89
Extracellular water, kg	15.74 ± 1.63^∗∗^	12.58 ± 1.89	13.18 ± 2.34^∗∗^	10.61 ± 1.72	11.24 ± 1.29^∗^	10.24 ± 1.10	13.28 ± 2.93^∗^	11.62 ± 1.88
Total energy expenditure, kcal	2888.45 ± 256.21^∗∗^	2571.34 ± 321.70	2537.35 ± 360.84^∗^	2189.62 ± 349.52	2191.13 ± 338.77	2136.06 ± 261.77	2477.95 ± 523.93	2406.17 ± 340.54
Basal metabolism (rest), kcal	1739.49 ± 148.07^∗∗^	1564.01 ± 174.09	1547.76 ± 217.00^∗^	1312.47 ± 243.86	1371.93 ± 102.37	1258.30 ± 164.21	1527.35 ± 230.24	1435.39 ± 199.36

Comparisons were performed between the patients with acromegaly and the controls. ^∗^*p* < 0.05; ^∗∗^*p* < 0.01.

**Table 3:** Body composition and metabolic changes in the acromegaly patients according to gender.

	Male			Female		
	Preoperative period	Postoperative period (3 months)	Postoperative period (1 year)	Preoperative period	Postoperative period (3 months)	Postoperative period (1 year)
Bicep circumference, cm	30.26 ± 1.93	31.25 ± 3.16	32.03 ± 2.85	27.96 ± 3.99	28.42 ± 3.01	27.78 ± 2.46
Tricep skinfold thickness, mm	18.94 ± 5.69	29.50 ± 10.78^∗∗^	20.20 ± 10.38	23.10 ± 8.95	28.08 ± 9.65	22.67 ± 8.50
Calf circumference, cm	39.11 ± 2.35	39.19 ± 2.11	39.32 ± 3.78	35.80 ± 2.42	37.15 ± 2.03	35.00 ± 2.19
Waistline, cm	89.48 ± 7.40	94.21 ± 9.44	99.37 ± 11.16^∗^	80.87 ± 8.27	85.44 ± 10.67	80.68 ± 6.73
Hipline, cm	102.94 ± 4.84	103.76 ± 6.15	107.38 ± 7.91	97.96 ± 5.96	100.31 ± 5.79	99.30 ± 2.35
Adipose tissue, kg	15.93 ± 4.40	16.65 ± 4.48	23.54 ± 12.17	18.79 ± 4.93	19.62 ± 6.08	17.23 ± 4.43
Visceral fat index	7.92 ± 1.78	8.97 ± 1.60	8.20 ± 0.60	9.39 ± 2.31	10.00 ± 2.94	8.75 ± 1.82
Sclerotin, kg	4.02 ± 0.32	3.91 ± 0.26	3.51 ± 0.52^∗∗^	3.00 ± 0.26	2.97 ± 0.29	2.68 ± 0.33^∗^
Protein, kg	13.23 ± 1.25	12.79 ± 0.94	12.81 ± 1.28	9.32 ± 1.01	9.39 ± 0.64	9.13 ± 0.87
Skeletal muscle, kg	42.94 ± 4.09	41.61 ± 3.55	36.54 ± 3.84^∗∗^	30.86 ± 4.40	30.61 ± 3.44	25.55 ± 2.57^∗^
Total body water, kg	46.93 ± 4.47	45.68 ± 3.54	47.31 ± 4.69	33.05 ± 3.63	33.41 ± 2.18	33.95 ± 3.12
Intracellular water, kg	31.19 ± 2.89	30.31 ± 2.38	29.80 ± 2.91	21.80 ± 2.37	22.02 ± 1.53	21.12 ± 1.97
Extracellular water, kg	15.74 ± 1.63	15.43 ± 1.61	17.80 ± 1.86^∗∗^	11.24 ± 1.29	11.37 ± 0.83	12.83 ± 1.18^∗^
Total energy expenditure, kcal	2888.45 ± 256.21	2822.78 ± 217.40	2461.04 ± 684.30	2191.13 ± 338.77	2269.66 ± 237.55	1751.29 ± 422.59^∗^
Basal metabolism (rest), kcal	1739.49 ± 148.07	1750.03 ± 164.93	1473.23 ± 502.99	1371.93 ± 102.37	1385.78 ± 135.66	998.97 ± 297.02^∗^

Comparisons were performed between the preoperative and postoperative periods. ^∗^*p* < 0.05; ^∗∗^*p* < 0.01.

**Table 4:** Body composition and metabolic changes in the acromegaly patients according to age.

	Age ≤ 40			Age > 40		
	Preoperative period	Postoperative period (3 months)	Postoperative period (1 year)	Preoperative period	Postoperative period (3 months)	Postoperative period (1 year)
Bicep circumference, cm	27.29 ± 3.01	27.94 ± 3.12	29.31 ± 3.20	30.06 ± 3.25	31.47 ± 2.65	31.56 ± 3.37
Tricep skinfold thickness, mm	18.25 ± 6.11	26.08 ± 9.40^∗^	17.25 ± 5.82	23.16 ± 8.37	31.23 ± 10.29^∗^	25.00 ± 11.19
Calf circumference, cm	36.57 ± 2.65	37.59 ± 2.27	36.27 ± 2.53	33.72 ± 2.99	38.63 ± 2.26	39.13 ± 4.56
Waistline, cm	82.36 ± 9.22	84.96 ± 9.07	89.29 ± 10.35	86.18 ± 8.59	93.98 ± 10.85^∗^	95.44 ± 15.84^∗^
Hipline, cm	98.52 ± 5.20	100.31 ± 5.40	102.35 ± 5.49	101.22 ± 6.30	103.49 ± 6.50	106.35 ± 8.95
Adipose tissue, kg	15.44 ± 3.57	16.02 ± 5.13	19.20 ± 7.84	18.84 ± 5.16	20.20 ± 5.17	23.15 ± 12.46
Visceral fat index	7.78 ± 1.66	8.52 ± 2.22	8.19 ± 0.69	9.34 ± 2.30	10.42 ± 2.27	8.63 ± 1.55
Sclerotin, kg	3.45 ± 0.53	3.41 ± 0.45	3.21 ± 0.66	3.45 ± 0.63	3.44 ± 0.64	3.19 ± 0.60
Protein, kg	11.06 ± 2.04	10.90 ± 1.79	11.44 ± 2.14	11.05 ± 2.44	11.14 ± 2.06	11.43 ± 2.30
Skeletal muscle, kg	35.89 ± 6.68	35.09 ± 5.62	32.40 ± 6.35	36.45 ± 7.97	36.62 ± 7.49	32.44 ± 6.93
Total body water, kg	39.22 ± 7.28	38.98 ± 6.78	42.10 ± 7.56	39.21 ± 8.66	39.59 ± 7.22	42.50 ± 8.57
Intracellular water, kg	26.04 ± 4.98	25.73 ± 4.42	26.68 ± 5.08	25.93 ± 5.75	26.25 ± 5.02	26.41 ± 5.31
Extracellular water, kg	13.18 ± 2.34	13.06 ± 2.28	15.79 ± 2.78^∗^	13.28 ± 2.93	13.55 ± 2.59	16.09 ± 3.29^∗^
Total energy expenditure, kcal	2537.35 ± 360.84	2519.80 ± 362.77	1889.69 ± 401.37^∗∗^	2477.95 ± 523.93	2549.32 ± 371.15	2500.08 ± 790.87
Basal metabolism (rest), kcal	1547.76 ± 217.00	1554.05 ± 222.97	1138.79 ± 359.31^∗^	1527.35 ± 230.24	1566.68 ± 258.21	1476.98 ± 559.73

Comparisons were performed between the preoperative and postoperative periods. ^∗^*p* < 0.05; ^∗∗^*p* < 0.01.
